# Supplementary material for: Independent control of the thermodynamic and kinetic properties of aptamer switches
Source: Nat Commun. 2019 Nov 7;10:5079. doi: 10.1038/s41467-019-13137-x (PMC6838323; doi:10.1038/s41467-019-13137-x)
Supplement: Supplementary file 1 — Supplementary Information [file 41467_2019_13137_MOESM1_ESM.pdf]

Supplementary Information

Independent Control of the Thermodynamic and Kinetic Properties of Aptamer Switches

Brandon D. Wilson et al.

Contents

Supplementary Figure 1 | Raw fluorescence traces and specificity test
Supplementary Figure 2 | Summary of thermodynamic and kinetic results
Supplementary Figure 3 | Two-site induced fit model
Supplementary Figure 4 | Conformational selection model
Supplementary Figure 5 | Binding curves for all ISD constructs
Supplementary Figure 6 | Binding curves for duplexed aptamers (unlinked displacement strands)
Supplementary Figure 7 | Additional mismatch results

Supplementary Table 1 | DNA sequences used
Supplementary Table 2 | Equivalence of linker and displacement strand lengths on effective binding affinity

Supplementary Note 1 | Relative effects of linker length and displacement strand length on effective binding affinity
Supplementary Note 2 | Derivation of signaling kinetics for induced fit
Supplementary Note 3 | Discussion of the limitations that the kinetics of the native aptamer impose on the kinetics of the ISD switch

Supplementary Figures

a)

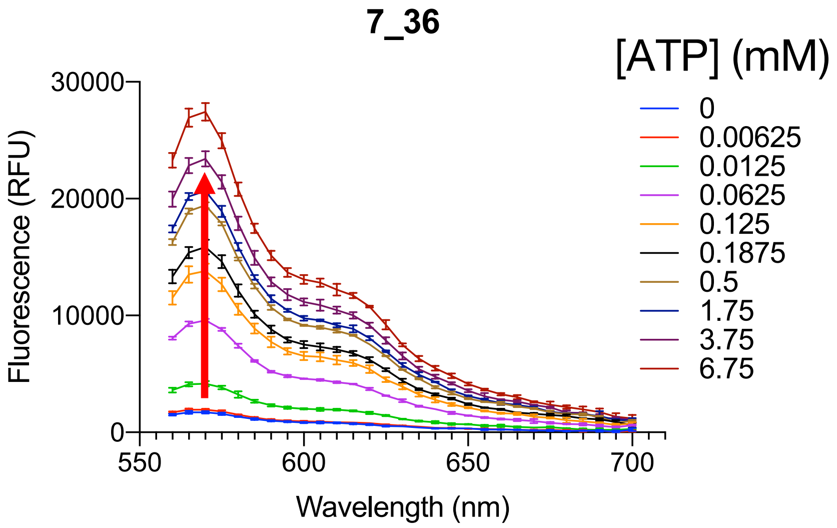

b)

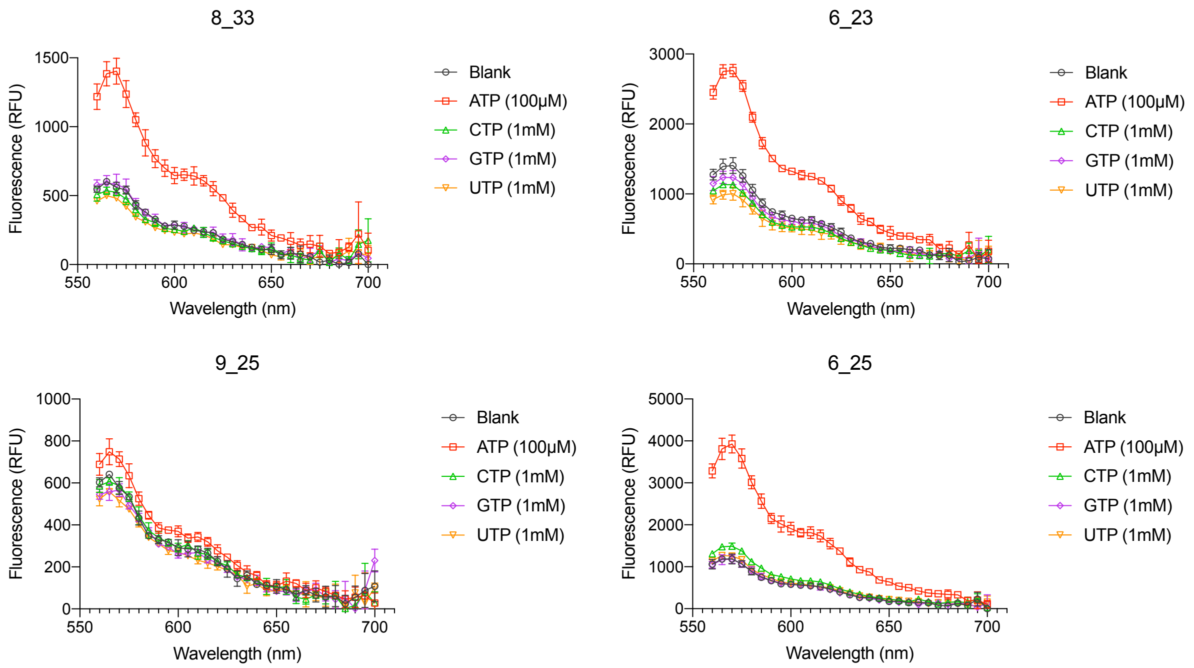

Supplementary Figure 1 | Raw signal analysis and selectivity of constructs. (a) Representative concentration-dependent emission spectra of an ISD switch. The fluorescence at peak emission (570 nm) was used as raw signal for both thermodynamic and kinetic studies. Concentrations of ATP in mM are listed. **(b)** Constructs with linker and double-stranded regions of varying lengths retain the high selectivity of the native aptamer for 100 μM ATP versus a 10-fold higher concentration of non-target ribonucleotides. All plots are averaged over n=3 replicates. Error bars represent the standard deviation. Source data are provided as a Source Data file.

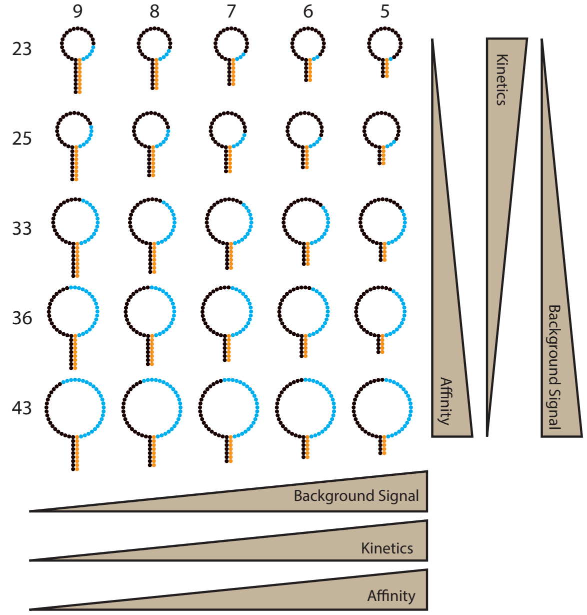

		K_D^{eff} (mM)			k_{obs}^{fast} (s^{-1})		
L_{DS}	L_{loop}	Average		Std. Err.	Best Fit	Upper CI	Lower CI
9	23	39.0	±	35.7	2.29	2.86	1.71
	25	12.8	±	1.7	1.70	1.94	1.45
	33	4.98	±	0.53	0.32	0.33	0.31
	36	2.72	±	1.47	0.31	0.32	0.29
	43	0.967	±	0.085	0.27	0.30	0.25
8	23	3.58	±	1.37	3.58	N/A	N/A
	25	2.29	±	0.05	3.61	4.02	3.21
	33	0.734	±	0.01	1.51	1.56	1.47
	36	0.352	±	0.043	1.77	1.85	1.69
	43	0.388	±	0.124	1.43	1.48	1.39
7	23	0.382	±	0.034	4.30	4.99	3.61
	25	0.271	±	0.038	4.02	4.43	3.60
	33	0.0985	±	0.0481	3.21	3.39	3.04
	36	0.0709	±	0.0018	2.95	3.18	2.73
	43	0.0298	±	0.0031	2.73	2.90	2.56
6	23	0.0314	±	0.0111	5.82	9.17	2.47
	25	0.0262	±	0.0095	4.18	4.71	3.65
	33	0.0182	±	0.0076	4.53	5.25	3.82
	36	0.0107	±	0.0019	4.82	5.65	3.99
	43	0.0149	±	0.0122	3.31	3.72	2.90
5	23	21.4	±	51.3	N/A	N/A	N/A
	25	0.0379	±	0.0191	N/A	N/A	N/A
	33	9.2	±	6.35	N/A	N/A	N/A
	36	0.0195	±	0.0538	N/A	N/A	N/A
	43	2.85	±	16.08	N/A	N/A	N/A

Supplementary Figure 2 | Overview of results for various ISD constructs in this work. The constructs tested are graphically summarized (left). The table (right) summarizes all measured thermodynamic and kinetic parameters for these constructs. For constructs where $L_{DS} = 5$ bp, kinetics were faster than the time resolution of our detector, and we could not obtain a robust fit. Affinities are reported as averages over n = 3 replicates ± the standard error calculated via propagation of errors (methods). Kinetic parameters were derived from fits to the normalized kinetic response averaged over n = 3 replicates. Variance in kinetic parameters is provided as the upper and lower 95% confidence intervals in the fit parameter. Source data are provided as a Source Data file.

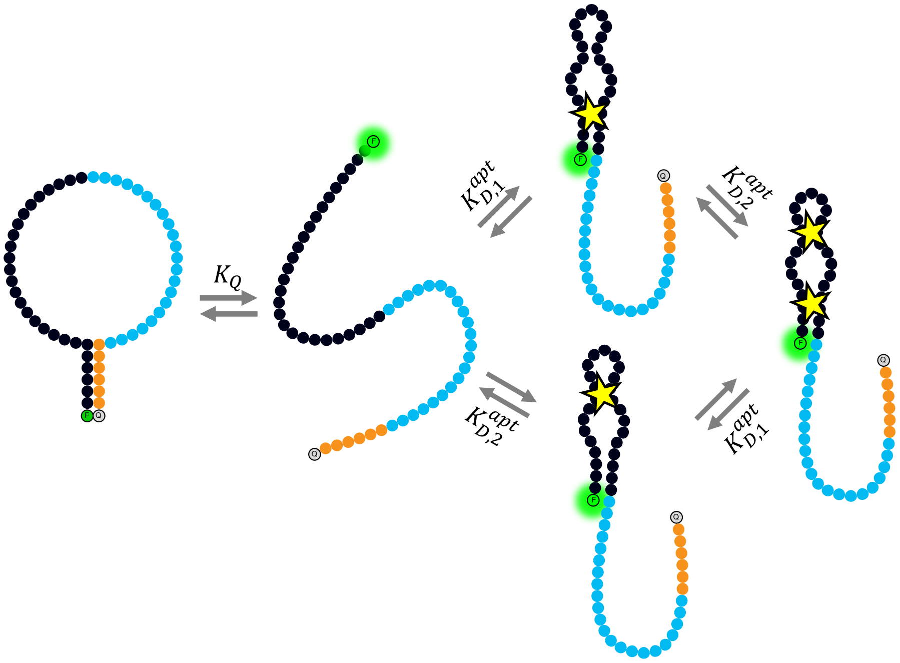

Supplementary Figure 3 | Two-site binding model. Here we derive the equations governing a two-site binding ISD construct. This model is specific to the ATP aptamer[1,2] and is likely to not be generalizable to other aptamers.

We recover the bimodal K_{D} behavior by allowing site 1 and site 2 to vary in fluorescence values. Without this assumption, the above model would result in a binding curve with a concave second derivative. We assume binding to site 1 and binding to site 2 have different fluorescence values, η_1 and η_2. We extract $K_{\mathrm{D},1}$ and K_{Q} via:

$$\text{Signal} = B_{\max} \frac{K_{\mathrm{D},1}K_{\mathrm{D},2}+\eta_1 K_{\mathrm{D},1}[T]+\eta_2 K_{\mathrm{D},2}[T]+K_{\mathrm{D},1}K_{\mathrm{D},2}[T]^2}{K_{\mathrm{D},1}K_{\mathrm{D},2}(1+K_{\mathrm{Q}})+K_{\mathrm{D},1}[T]+K_{\mathrm{D},2}[T]+K_{\mathrm{D},1}K_{\mathrm{D},2}[T]^2} \tag{1}$$

In the manuscript, we report $K_{\mathrm{D}}^{\mathrm{eff}}$ as calculated by Equation (4) using $K_{\mathrm{D},2}$.

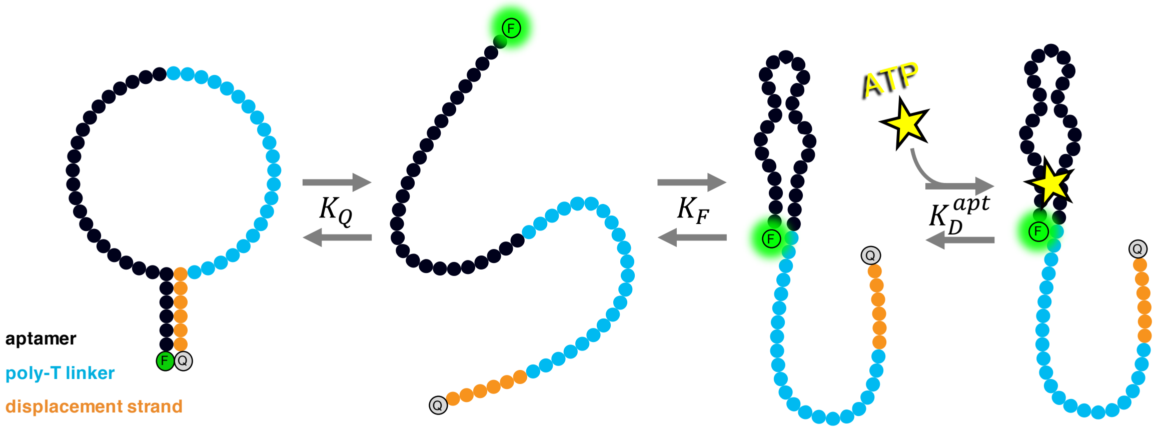

Supplementary Figure 4 | Conformational selection model. It has been suggested that the ATP aptamer used in this work can undergo both induced-fit[3] and conformational selection[4] binding mechanisms. Therefore, we present the equations governing ISD through a single-site, conformational selection binding mechanism. Since the pre-folded, binding-competent form also gives a signal, aptamers exhibiting this binding mechanism are more likely to suffer from larger background signal or possibly slower kinetics[5].

$$\text{Signal} = \frac{1+K_{\mathrm{F}}+\frac{K_{\mathrm{F}}[T]}{K_{\mathrm{D}}^{\mathrm{apt}}}}{K_{\mathrm{Q}}+1+K_{\mathrm{F}}+\frac{K_{\mathrm{F}}[T]}{K_{\mathrm{D}}^{\mathrm{apt}}}} \quad (2)$$

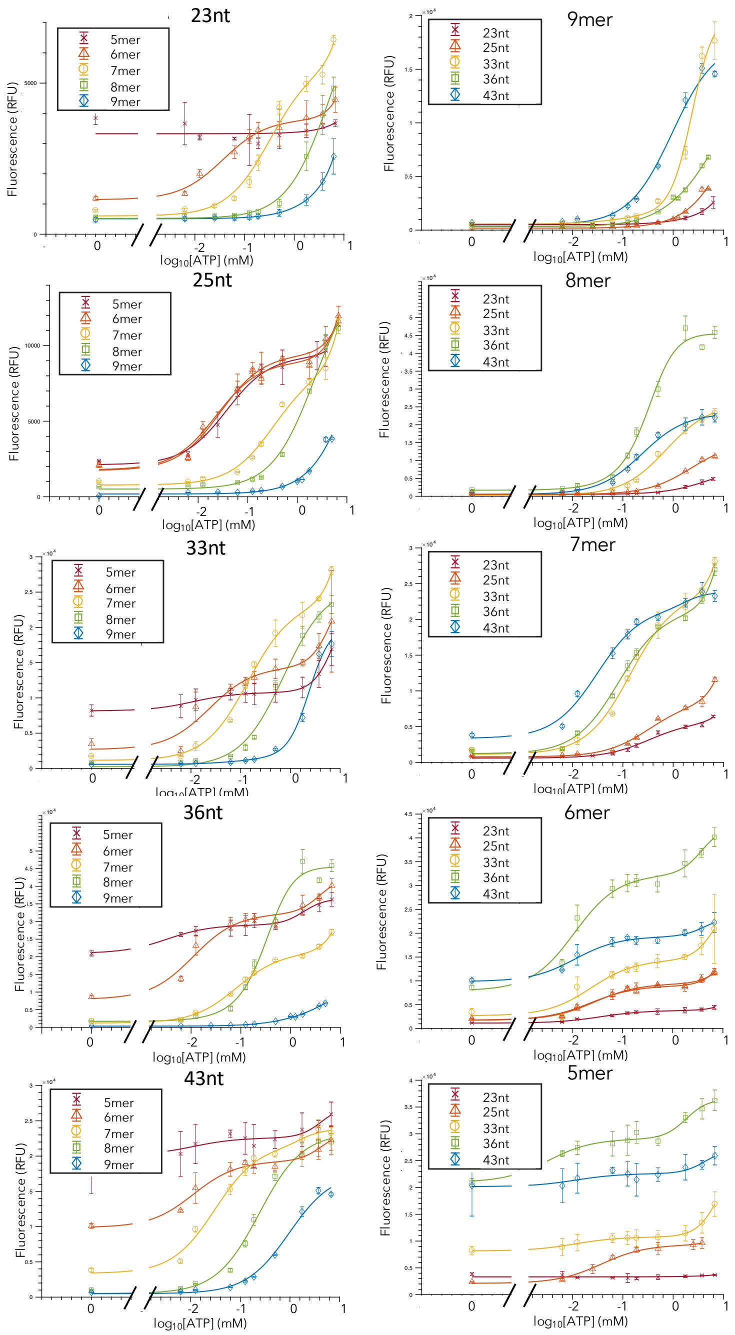

Supplementary Figure 5 | Binding curves for all ISD constructs. Plots show raw fluorescence as a function of [ATP] for different ISD constructs averaged over n=3 replicates. Error bars represent the standard deviation. Source data are provided as a Source Data file.

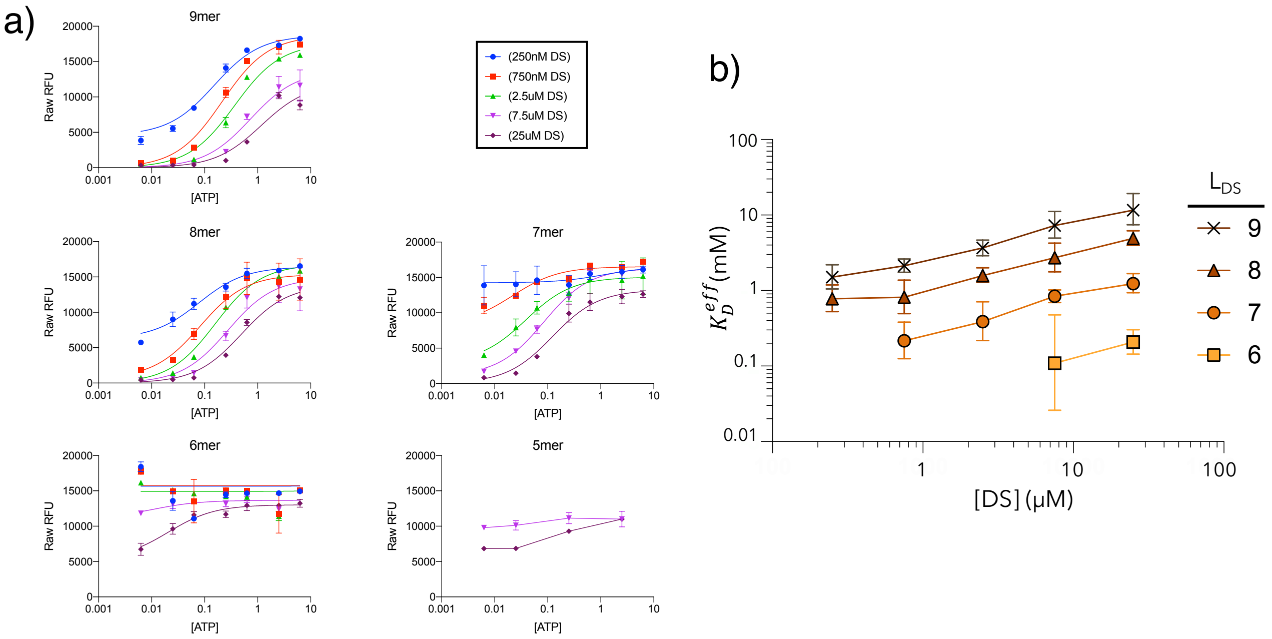

Supplementary Figure 6 | Binding curves for duplexed aptamers (DA). (**a**) plots of duplexed aptamers (i.e. unlinked displacement strand) with displacement strands of various lengths and concentrations. Varying the concentration of the DS simulates the change in effective concentration that occurs due to changes in linker length. (**b**) Fits of $K_{\mathrm{D}}^{\mathrm{eff}}$ to data in **a**. As concentration increases, the equilibrium shifts towards the quenched state, which increases the effective binding affinity. Aptamer concentration is 250 nM. All plots represent triplicate experiments. The error bars in plot **a** represent the standard deviation (n=3) whereas error bars in plot **b** represent the 95% confidence interval of the fit to a single-site binding curve. Source data are provided as a Source Data file.

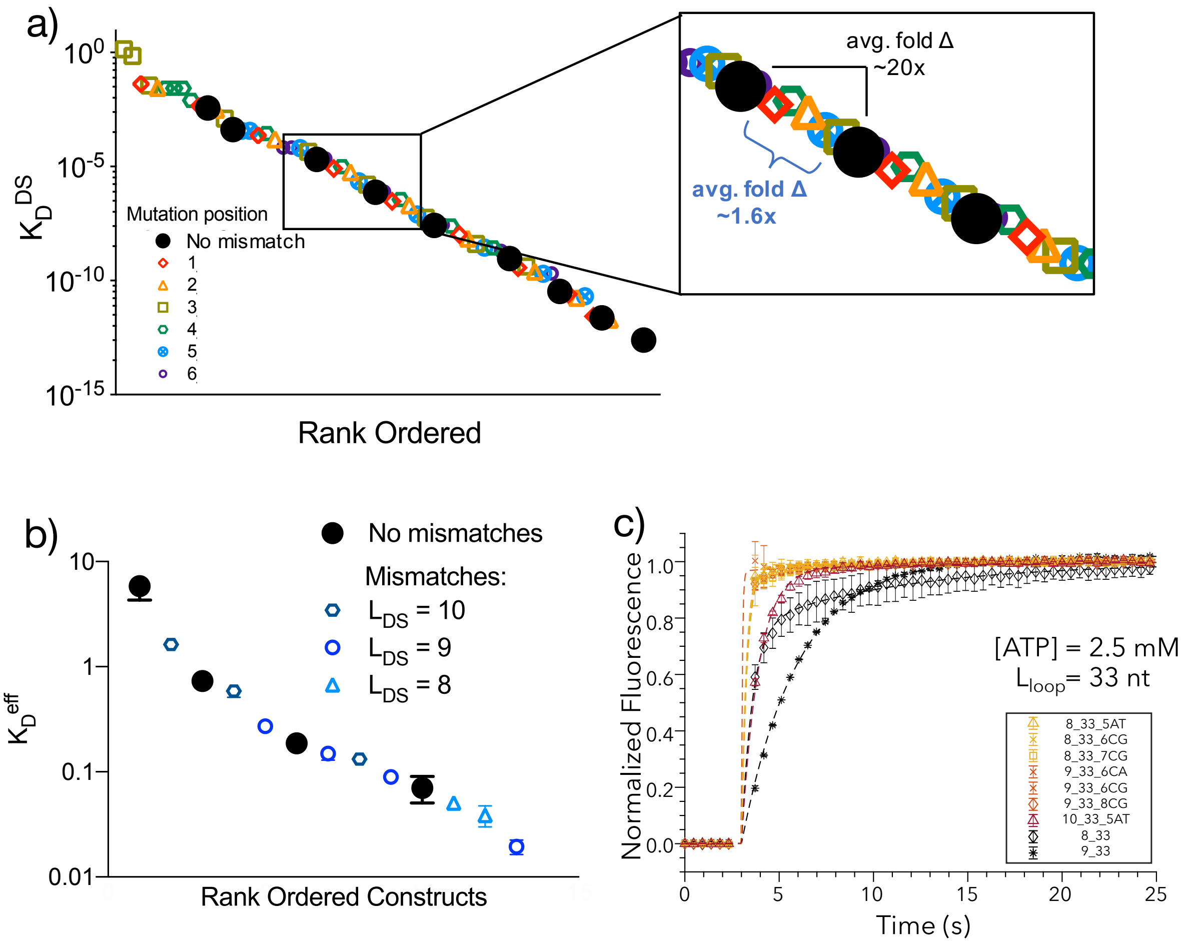

Supplementary Figure 7 | Effects of mismatch incorporation. (**a**) When considering perfectly matched, unlinked displacement strands (black circles) the set of theoretically obtainable K_D^{DS} features very large jumps (20.6 ± 8.7 per base). On the other hand, incorporating single mismatches (colored symbols) greatly decreases the average fold spacing between obtainable K_D^{DS} to 1.6 ± 0.8. Calculations were performed using ΔG_{fold} from *mfold* at room temperature and 6 mM Mg^{2+}. Mutation position is defined from the 3'-end of the displacement strand. (**b**) Measured effective binding affinities for perfectly matched strands (black circles) and mismatches (colored symbols) with L_{loop} = 33 nt. Adding mismatches greatly increases the tunability of the thermodynamics. (**c**) Introducing mismatches greatly increases the signaling kinetics of the constructs, e.g. yellow vs. black star and orange vs. black diamond. Even a 10mer DS with a single mismatch (red) exhibits kinetics faster than that of the corresponding aptamer beacon[6]. Plots **b** and **c** are averages over n=3. Error bars in **b** are calculated according to propagation of errors, and the error bars in **c** are the standard deviation. Source data are provided as a Source Data file.

Supplementary Tables

Supplementary Table 1 | Sequences used in this work. Black text represents the aptamer sequence, blue text represents the poly-T linker, and orange text represents the displacement strand sequence. Underlined sequence is complementary to the displacement strand. L_{loop} is equal to the number of bases between the underlined region and the displacement strand. The loop length in this design cannot be shorter than that of the aptamer minus the length of the displacement strand (in this case, 23 nt). Mismatches introduced into the ISD constructs are shown in red.

	L_{DS}	L_{loop}	Sequence 5' --> 3'	Total Length	Poly T
Perfect match	10	33	CACCTGGGGGAGTATTGCGGAGGAAGGTTTTTTTTTTTTTTTTCCCCCAGGTG	53	16
	9	23	CACCTGGGGGAGTATTGCGGAGGAAGGTTTTTCCCCAGGTG	41	5
	9	25	CACCTGGGGGAGTATTGCGGAGGAAGGTTTTTTTCCCCAGGTG	43	7
	9	33	CACCTGGGGGAGTATTGCGGAGGAAGGTTTTTTTTTTTTTTTCCCCAGGTG	51	15
	9	36	CACCTGGGGGAGTATTGCGGAGGAAGGTTTTTTTTTTTTTTTTTTCCCCAGGTG	54	18
	9	43	CACCTGGGGGAGTATTGCGGAGGAAGGTTTTTTTTTTTTTTTTTTTTTTTTTCCCCAGGTG	61	25
	8	23	CACCTGGGGGAGTATTGCGGAGGAAGGTTTTCCCAGGTG	39	4
	8	25	CACCTGGGGGAGTATTGCGGAGGAAGGTTTTTTCCCAGGTG	41	6
	8	33	CACCTGGGGGAGTATTGCGGAGGAAGGTTTTTTTTTTTTTTCCCAGGTG	49	14
	8	36	CACCTGGGGGAGTATTGCGGAGGAAGGTTTTTTTTTTTTTTTTTCCCAGGTG	52	17
	8	43	CACCTGGGGGAGTATTGCGGAGGAAGGTTTTTTTTTTTTTTTTTTTTTTTTCCCAGGTG	59	24
	7	23	CACCTGGGGGAGTATTGCGGAGGAAGGTTTCCAGGTG	37	3
	7	25	CACCTGGGGGAGTATTGCGGAGGAAGGTTTTTCCAGGTG	39	5
	7	33	CACCTGGGGGAGTATTGCGGAGGAAGGTTTTTTTTTTTTTCCAGGTG	47	13
	7	36	CACCTGGGGGAGTATTGCGGAGGAAGGTTTTTTTTTTTTTTTTCCAGGTG	50	16
	7	43	CACCTGGGGGAGTATTGCGGAGGAAGGTTTTTTTTTTTTTTTTTTTTTTTCCAGGTG	57	23
	6	23	CACCTGGGGGAGTATTGCGGAGGAAGGTTCAGGTG	35	2
	6	25	CACCTGGGGGAGTATTGCGGAGGAAGGTTTTCAGGTG	37	4
	6	33	CACCTGGGGGAGTATTGCGGAGGAAGGTTTTTTTTTTTTCAGGTG	45	12
	6	36	CACCTGGGGGAGTATTGCGGAGGAAGGTTTTTTTTTTTTTTTCAGGTG	48	15
	6	43	CACCTGGGGGAGTATTGCGGAGGAAGGTTTTTTTTTTTTTTTTTTTTTTCAGGTG	55	22
	5	23	CACCTGGGGGAGTATTGCGGAGGAAGGTAGGTG	33	1
	5	25	CACCTGGGGGAGTATTGCGGAGGAAGGTTTAGGTG	35	3
	5	33	CACCTGGGGGAGTATTGCGGAGGAAGGTTTTTTTTTTTAGGTG	43	11
	5	36	CACCTGGGGGAGTATTGCGGAGGAAGGTTTTTTTTTTTTTTAGGTG	46	14
	5	43	CACCTGGGGGAGTATTGCGGAGGAAGGTTTTTTTTTTTTTTTTTTTTTAGGTG	53	21
Mismatches	10	33	CACCTGGGGGAGTATTGCGGAGGAAGGTTTTTTTTTTTTTTTTCGCCCAGGTG	53	16
	10	33	CACCTGGGGGAGTATTGCGGAGGAAGGTTTTTTTTTTTTTTTTCCCGCAGGTG	53	16
	10	33	CACCTGGGGGAGTATTGCGGAGGAAGGTTTTTTTTTTTTTTTTCCCCCTGGTG	53	16
	10	33	CACCTGGGGGAGTATTGCGGAGGAAGGTTTTTTTTTTTTTTTTCCCCCGGGTG	53	16
	9	33	CACCTGGGGGAGTATTGCGGAGGAAGGTTTTTTTTTTTTTTTCGCCAGGTG	51	15
	9	33	CACCTGGGGGAGTATTGCGGAGGAAGGTTTTTTTTTTTTTTTCTCCAGGTG	51	15
	9	33	CACCTGGGGGAGTATTGCGGAGGAAGGTTTTTTTTTTTTTTTCACCAGGTG	51	15
	9	33	CACCTGGGGGAGTATTGCGGAGGAAGGTTTTTTTTTTTTTTTCCGCAGGTG	51	15
	9	33	CACCTGGGGGAGTATTGCGGAGGAAGGTTTTTTTTTTTTTTTCCCGAGGTG	51	15
	9	33	CACCTGGGGGAGTATTGCGGAGGAAGGTTTTTTTTTTTTTTTCCCTAGGTG	51	15
	9	33	CACCTGGGGGAGTATTGCGGAGGAAGGTTTTTTTTTTTTTTTCCCAAGGTG	51	15
	8	33	CACCTGGGGGAGTATTGCGGAGGAAGGTTTTTTTTTTTTTTCGCAGGTG	49	14
	8	33	CACCTGGGGGAGTATTGCGGAGGAAGGTTTTTTTTTTTTTTCCGAGGTG	49	14
	8	33	CACCTGGGGGAGTATTGCGGAGGAAGGTTTTTTTTTTTTTTCCCTGGTG	49	14
	8	33	CACCTGGGGGAGTATTGCGGAGGAAGGTTTTTTTTTTTTTTCCCACGTG	49	14

Supplementary Table 2 | Equivalence of loop and DS lengths on effective binding affinity. Loop and displacement strand lengths have different per base effects on effective binding affinity (Supplementary Note 1), i.e. adding a single base to the displacement strand should have a greater impact on the effective binding affinity than removing a single base from the linker. Here we examine the change in loop length required to cause the same change in $K_{\mathrm{D}}^{\mathrm{eff}}$ as the addition of one base to the displacement strand, $\frac{\Delta L_{\mathrm{loop}}}{\Delta L_{\mathrm{DS}}}$. Errors represent the propagation of errors for the ratio of slopes of linear fits to Figures 3b and 3d, as described in Supplementary Note 1. Initial data used n=3 replicates. Because of their imprecision, values for $K_{\mathrm{D}}^{\mathrm{eff}}$ corresponding to $L_{\mathrm{DS}} = 5$ bp were omitted when calculating $\left.\frac{\mathrm{d}\log K_{\mathrm{Q}}}{\mathrm{d}L_{\mathrm{DS}}}\right|_{\mathrm{constant}\ L_{\mathrm{loop}}}$. Source data are provided as a Source Data file.

$\frac{\Delta L_{\mathrm{loop}}}{\Delta L_{\mathrm{DS}}}$		L_{loop}				
		23	25	33	36	43
L_{DS}	5	-94.9 ± 863.8	-83.0 ± 756.2	-75.7 ± 689.6	-73.2 ± 666.4	-55.3 ± 503.6
	6	-53.3 ± 20.3	-46.7 ± 17.9	-42.6 ± 16.2	-41.2 ± 15.7	-31.1 ± 13.1
	7	-18.7 ± 0.5	-16.4 ± 0.9	-15.0 ± 0.5	-14.5 ± 0.6	-10.9 ± 2.0
	8	-13.5 ± 0.6	-11.8 ± 0.7	-10.8 ± 0.5	-10.4 ± 0.5	-7.9 ± 1.5
	9	-13.9 ± 1.6	-12.2 ± 1.5	-11.1 ± 1.3	-10.7 ± 1.3	-8.1 ± 1.7

Supplementary Notes

Supplementary Note 1 | The relative impacts of L_{loop} and L_{DS} on K_{Q}. We can calculate the equivalence between the parameters as follows:

$$K_{\text{Q}} \sim \frac{\exp\left(\frac{1.7 L_{\text{DS}}}{RT}\right)}{\left(L_{\text{loop}}\right)^{2.6}} \quad (3)$$

$$\frac{\mathrm{d}K_{\text{Q}}}{\mathrm{d}L_{\text{DS}}} \sim \frac{1.7}{RT} K_{\text{Q}} \quad (4)$$

$$\frac{\mathrm{d}K_{\text{Q}}}{\mathrm{d}L_{\text{loop}}} \sim -\frac{2.6}{L_{\text{loop}}} K_{\text{Q}} \quad (5)$$

At room temperature, we find:

$$\frac{\mathrm{d}K_{\text{Q}}}{\mathrm{d}L_{\text{loop}}} \sim \frac{-7.5}{L_{\text{loop}}} \frac{\mathrm{d}K_{\text{Q}}}{\mathrm{d}L_{\text{DS}}} \quad (6)$$

Fits to Figure 3b and d yield $\frac{L_{\text{loop}} \frac{\mathrm{d}K_{\text{Q}}}{\mathrm{d}L_{\text{loop}}}}{\frac{\mathrm{d}K_{\text{Q}}}{\mathrm{d}L_{\text{DS}}}} = -6.0 \pm 3.4$, which is in agreement with our theory.

More specifically, linear fits to Figure 3b and d yield:

$$a = \left.\frac{\mathrm{d}\log K_{\text{Q}}}{\mathrm{d}L_{\text{loop}}}\right|_{\text{constant } L_{\text{DS}}} = -0.049 \pm 0.030 \ \frac{\text{log change } K_{\text{Q}}}{\text{loop nt}}$$

$$b = \left.\frac{\mathrm{d}\log K_{\text{Q}}}{\mathrm{d}L_{\text{DS}}}\right|_{\text{constant } L_{\text{loop}}} = 0.827 \pm 0.154 \ \frac{\text{log change } K_{\text{Q}}}{\text{DS bp}}$$

Where σ = standard deviation over the five combinations of linker or DS.
The equivalence between loop bases and displacement strand bases is then given by:

$$\frac{\Delta L_{\text{loop}}}{\Delta L_{\text{DS}}} = \frac{\left.\frac{\mathrm{d}\log K_{\text{Q}}}{\mathrm{d}L_{\text{DS}}}\right|_{\text{constant } L_{\text{loop}}}}{\left.\frac{\mathrm{d}\log K_{\text{Q}}}{\mathrm{d}L_{\text{loop}}}\right|_{\text{constant } L_{\text{DS}}}} = -17.7 \pm 11.9$$

Where σ is calculated using propagation of errors: $\sigma = \sqrt{\left(\frac{\sigma_b}{a}\right)^2 + \left(\frac{\text{b}\,\sigma_a}{a^2}\right)^2}$

The loop/DS equivalence varies over L_{DS} and L_{loop} and is summarized in Supplementary Table 2.

Supplementary Note 2 | Derivation of the signaling kinetics of induced fit binding. Kinetics for induced fit can be derived[7] as follows (see Mathematica code at https://github.com/btotherad77/isd for full analytical solution). Importantly, the analysis reveals two effective time constants—a fast time constant and a slow time constant—that are functions of the parameters of both aptamer and displacement strand kinetics. Since we assume the kinetics of aptamer binding are fixed, we obtain control of k_{obs} by modulating $k_{\mathrm{on}}^{\mathrm{DS}}$ and $k_{\mathrm{off}}^{\mathrm{DS}}$.

$$\mathbf{C}(t) = \begin{pmatrix} C_{\mathrm{Q}}(t) \\ C_{\mathrm{F}}(t) \\ C_{\mathrm{B}}(t) \end{pmatrix} \tag{7}$$

$$\mathbf{k} = \begin{pmatrix} -k_{\mathrm{off}}^{\mathrm{DS}} & k_{\mathrm{on}}^{\mathrm{DS}} & 0 \\ k_{\mathrm{off}}^{\mathrm{DS}} & -\left(k_{\mathrm{off}}^{\mathrm{DS}} + k_{\mathrm{on}}^{\mathrm{apt}}[T]\right) & k_{\mathrm{off}}^{\mathrm{apt}} \\ 0 & k_{\mathrm{on}}^{\mathrm{apt}}[T] & -k_{\mathrm{off}}^{\mathrm{apt}} \end{pmatrix} \tag{8}$$

$$\frac{\mathrm{d}}{\mathrm{d}t}\mathbf{C}(t) = \mathbf{k} \bullet \mathbf{C}(t) \tag{9}$$

$$\mathbf{C}(t) = \mathbf{x} \exp(\mathbf{\Lambda} t)\, \mathbf{x}^{-1} \bullet \mathbf{C}_0 \tag{10}$$

Where x is the matrix of eigen vectors of k, and lambda represents the diagonal matrix of eigenvalues such that

$$\exp(\mathbf{\Lambda} t) = \begin{pmatrix} \exp(\lambda_0 t) & \cdots & 0 \\ \vdots & \ddots & \vdots \\ 0 & \cdots & \exp(\lambda_{\mathrm{n}} t) \end{pmatrix}. \tag{11}$$

This system of equations can be solved to yield two effective rate constants:

$$k_{\mathrm{obs}}^{\mathrm{fast}} = \frac{1}{2}\Bigg(k_{\mathrm{off}}^{\mathrm{DS}} + k_{\mathrm{on}}^{\mathrm{DS}} + [T]k_{\mathrm{on}}^{\mathrm{apt}} + k_{\mathrm{off}}^{\mathrm{apt}} + \sqrt{\left(k_{\mathrm{off}}^{\mathrm{DS}} + k_{\mathrm{on}}^{\mathrm{DS}} + [T]k_{\mathrm{on}}^{\mathrm{apt}} + k_{\mathrm{off}}^{\mathrm{apt}}\right)^2 - 4\left(k_{\mathrm{on}}^{\mathrm{DS}}k_{\mathrm{off}}^{\mathrm{apt}} + k_{\mathrm{off}}^{\mathrm{DS}}\left(k_{\mathrm{off}}^{\mathrm{apt}} + [T]k_{\mathrm{on}}^{\mathrm{apt}}\right)\right)}\Bigg) \tag{12}$$

$$k_{\mathrm{obs}}^{\mathrm{slow}} = \frac{1}{2}\Bigg(k_{\mathrm{off}}^{\mathrm{DS}} + k_{\mathrm{on}}^{\mathrm{DS}} + [T]k_{\mathrm{on}}^{\mathrm{apt}} + k_{\mathrm{off}}^{\mathrm{apt}} - \sqrt{\left(k_{\mathrm{off}}^{\mathrm{DS}} + k_{\mathrm{on}}^{\mathrm{DS}} + [T]k_{\mathrm{on}}^{\mathrm{apt}} + k_{\mathrm{off}}^{\mathrm{apt}}\right)^2 - 4\left(k_{\mathrm{on}}^{\mathrm{DS}}k_{\mathrm{off}}^{\mathrm{apt}} + k_{\mathrm{off}}^{\mathrm{DS}}\left(k_{\mathrm{off}}^{\mathrm{apt}} + [T]k_{\mathrm{on}}^{\mathrm{apt}}\right)\right)}\Bigg) \tag{13}$$

The implications of these two rate constants are discussed in Supplementary Note 3.

Supplementary Note 3 | The limitations imposed by the kinetics of the native aptamer. Like the thermodynamics, the kinetics of the native aptamer impose limits on the kinetic response of our ISD constructs. The observed kinetics of the ISD construct will always be an additive mixture of two exponential responses with fast and slow kinetic rates. Starting from our analytically determined rate constants for the induced fit model (Supplementary Note 2), we consider the two limiting cases where the kinetics of displacement strand binding have been tuned to be arbitrarily slower or faster than the kinetics of the native aptamer such that $k_{\text{off}}^{\text{DS}}, k_{\text{on}}^{\text{DS}} \ll k_{\text{off}}^{\text{apt}}, [T]k_{\text{on}}^{\text{apt}}$ or $k_{\text{off}}^{\text{DS}}, k_{\text{on}}^{\text{DS}} \gg k_{\text{off}}^{\text{apt}}, [T]k_{\text{on}}^{\text{apt}}$.

For the first case where the displacement strand kinetics have been engineered to be much slower than the aptamer kinetics, we have, for $k_{\text{off}}^{\text{DS}}, k_{\text{on}}^{\text{DS}} \ll k_{\text{off}}^{\text{apt}}, [T]k_{\text{on}}^{\text{apt}}$:

$$k_{\text{obs}}^{\text{fast}} = [T]k_{\text{on}}^{\text{apt}} + k_{\text{off}}^{\text{apt}} \tag{14}$$

And by applying the binomial approximation to Supplementary Equation (13)—justified since $4\left(k_{\text{on}}^{\text{DS}}k_{\text{off}}^{\text{apt}} + k_{\text{off}}^{\text{DS}}\left(k_{\text{off}}^{\text{apt}} + [T]k_{\text{on}}^{\text{apt}}\right)\right) \ll \left(k_{\text{off}}^{\text{DS}} + k_{\text{on}}^{\text{DS}} + [T]k_{\text{on}}^{\text{apt}} + k_{\text{off}}^{\text{apt}}\right)^2$—we obtain

$$k_{\text{obs}}^{\text{slow}} = k_{\text{off}}^{\text{DS}} + k_{\text{on}}^{\text{DS}}\frac{k_{\text{off}}^{\text{apt}}}{[T]k_{\text{on}}^{\text{apt}} + k_{\text{off}}^{\text{apt}}} \tag{15}$$

Therefore, in the case where the displacement strand kinetics have been engineered to be slow as compared to the aptamer, we can arbitrarily decrease the slow time constant, however the fast time constant is constrained by the native aptamer.

For the second case where we have engineered the displacement strand kinetics to be much faster than the aptamer binding kinetics, we have, for $k_{\text{off}}^{\text{DS}}, k_{\text{on}}^{\text{DS}} \gg k_{\text{off}}^{\text{apt}}, [T]k_{\text{on}}^{\text{apt}}$:

$$\text{k}_{\text{obs}}^{\text{fast}} = k_{\text{off}}^{\text{DS}} + k_{\text{on}}^{\text{DS}} \tag{16}$$

Again, applying the binomial approximation to Supplementary Equation (13):

$$k_{\text{obs}}^{\text{slow}} = k_{\text{off}}^{\text{apt}} + [T]k_{\text{on}}^{\text{apt}}\frac{k_{\text{off}}^{\text{DS}}}{k_{\text{on}}^{\text{DS}} + k_{\text{off}}^{\text{DS}}} \tag{17}$$

Therefore, in the case where the displacement strand kinetics have been engineered to be faster than the native aptamer, we can arbitrarily increase the fast time constant, while the slow time constant is constrained by the native aptamer.

The observed kinetics of the ISD construct will always be an additive mixture of two exponential responses with the slow and fast kinetic rates. The relative magnitude of these contributions is determined, assuming the system begins at equilibrium, by the initial and final target concentrations. From the above two results, we can see that in either limiting design case, one rate constant will be bounded by the native aptamer properties. The deconvolution of fast and slow responses is given by:

$$\mathbf{C}(\text{t}) = \mathbf{C}_{\text{eq}} + \mathbf{v}_{\text{slow}}\left(\mathbf{v}_{\text{slow}}^{-\mathbf{1}} \bullet \mathbf{C}_0\right)\text{e}^{-k_{\text{slow}}\boldsymbol{t}} + \mathbf{v}_{\text{fast}}\left(\mathbf{v}_{\text{fast}}^{-\mathbf{1}} \bullet \mathbf{C}_0\right)\text{e}^{-k_{\text{fast}}\boldsymbol{t}} \tag{18}$$

Where the eigenvectors* ($\boldsymbol{v}$) and eigenvalues* are set by the final equilibrium concentrations and $\mathbf{C}_0$ is the initial concentrations. Depending on the initial and final values of [T], the distribution of $k_{\text{obs}}^{\text{fast}}$ vs. $k_{\text{obs}}^{\text{slow}}$ can be changed via $\mathbf{v}_{\text{fast}}\left(\mathbf{v}_{\text{fast}}^{-\mathbf{1}} \bullet \mathbf{C}_0\right)$ and $\mathbf{v}_{\text{slow}}\left(\mathbf{v}_{\text{slow}}^{-\mathbf{1}} \bullet \mathbf{C}_0\right)$. Thus, one will always be able to pick some initial and final target concentrations to induce a binding response that is bounded by the kinetic properties of the native aptamer, regardless of ISD design. However, we note that for slow aptamer kinetics, if $\mathbf{v}_{\text{fast}}\left(\mathbf{v}_{\text{fast}}^{-\mathbf{1}} \bullet \mathbf{C}_0\right) \gg \mathbf{v}_{\text{slow}}\left(\mathbf{v}_{\text{slow}}^{-\mathbf{1}} \bullet \mathbf{C}_0\right)$ and $k_{\text{off}}^{\text{DS}}, k_{\text{on}}^{\text{DS}} \gg k_{\text{off}}^{\text{apt}}, [T]k_{\text{on}}^{\text{apt}}$, the observed response will be independent of native aptamer kinetics.

*the *mathematica* script with the analytical solutions to the eigenvalues and eigenvectors is provided (https://github.com/btotherad77/isd).

Supplementary References

1. Huizenga, D. E. & Szostak, J. W. A DNA Aptamer That Binds Adenosine and ATP. *Biochemistry* **34**, 656–665 (1995).
2. Zhang, Z., Oni, O. & Liu, J. New insights into a classic aptamer: Binding sites, cooperativity and more sensitive adenosine detection. *Nucleic Acids Res.* **45**, 7593–7601 (2017).
3. Munzar, J. D., Ng, A. & Juncker, D. Comprehensive profiling of the ligand binding landscapes of duplexed aptamer families reveals widespread induced fit. *Nat. Commun.* **9**, (2018).
4. Xia, T., Yuan, J. & Fang, X. Conformational dynamics of an ATP-binding DNA aptamer: A single-molecule study. *J. Phys. Chem. B* **117**, 14994–15003 (2013).
5. Spiridonova, V. A. *et al.* A family of DNA aptamers with varied duplex region length that forms complexes with thrombin and prothrombin. *FEBS Lett.* **589**, 2043–2049 (2015).
6. Nutiu, R. & Li, Y. Structure-switching signaling aptamers. *J. Am. Chem. Soc.* **125**, 4771–4778 (2003).
7. Vogt, A. D. & Di Cera, E. Conformational selection or induced fit? A critical appraisal of the kinetic mechanism. *Biochemistry* **51**, 5894–5902 (2012).
